# Complex Codon Usage Pattern and Compositional Features of Retroviruses

**DOI:** 10.1155/2013/848123

**Published:** 2013-10-31

**Authors:** Sourav RoyChoudhury, Debaprasad Mukherjee

**Affiliations:** ^1^School of Information Technology, Bengal Engineering and Science University, Shibpur, Howrah, West Bengal 711103, India; ^2^School of Medical Science and Technology, Indian Institute of Technology Kharagpur, Kharagpur-721302, India; ^3^Department of Information Technology, Dr. B.C. Roy Engineering College, West Bengal University of Technology, Durgapur, West Bengal 713206, India

## Abstract

Retroviruses infect a wide range of organisms including humans. Among them, HIV-1, which causes AIDS, has now become a major threat for world health. Some of these viruses are also potential gene transfer vectors. In this study, the patterns of synonymous codon usage in retroviruses have been studied through multivariate statistical methods on ORFs sequences from the available 56 retroviruses. The principal determinant for evolution of the codon usage pattern in retroviruses seemed to be the compositional constraints, while selection for translation of the viral genes plays a secondary role. This was further supported by multivariate analysis on relative synonymous codon usage. Thus, it seems that mutational bias might have dominated role over translational selection in shaping the codon usage of retroviruses. Codon adaptation index was used to identify translationally optimal codons among genes from retroviruses. The comparative analysis of the preferred and optimal codons among different retroviral groups revealed that four codons GAA, AAA, AGA, and GGA were significantly more frequent in most of the retroviral genes inspite of some differences. Cluster analysis also revealed that phylogenetically related groups of retroviruses have probably evolved their codon usage in a concerted manner under the influence of their nucleotide composition.

## 1. Introduction

The retroviruses are a diverse family of enveloped single stranded retro transcribing RNA viruses unique for their use of reverse transcription of the viral RNA into linear double stranded DNA during replication and the subsequent integration of the DNA into the host genome. Members of this family cause diseases in a wide range of organisms, including humans [1]. Human immunodeficiency virus 1 (HIV-1) is responsible for acquired immunodeficiency syndrome (AIDS) and is largely dependent on transmission of contaminated body fluids during sexual intercourse, pregnancy, and so forth [2]. More than 30 million people worldwide are living with HIV. Besides, retroviruses are increasingly becoming valuable tools in molecular biology and have been used successfully in gene therapy [3]. Based on morphology, pathogenicity, and molecular phylogenetics, retroviruses have been classified into two subfamilies: Orthoretrovirinae, Spumaretrovirinae and rest of the viruses are unclassified. The Orthoretrovirinae is further classified into six genera: *Alpharetrovirus, Betaretrovirus, Deltaretrovirus, Epsilonretrovirus, Gammaretrovirus,* and *Lentivirus*.

The analysis of codon usage of whole organisms and/or organisms from closely related groups of them reveals trends and anomalies in the choice and bias in the frequency of codons and related nucleotide composition, including evolutionary features. Synonymous codons do not occur in equal frequency in genes and genomes. The relative frequency of these synonymous codons in the genes varies significantly in a nonrandom manner between species, even between those from the same taxon due to a complex balance between mutational bias, various selection forces (e.g., translational selection), and drift acting on the genes or genomes [4]. Codon and base usage patterns reveal information on the nature of molecular evolution of genes and genomes, sometimes even events of horizontal gene transfer. Evidence exists of correlations between codon usage bias and nucleotide composition in some viruses, clearly indicating that mutational bias towards particular nucleotide content influences general codon usage of organisms [5]. For example, in free living organisms, such as *E. coli*, *S. cerevisiae, C. elegans, D. melanogaster*, and* A. thaliana*, knowledge of codon usage bias gives insights into the content of the isoacceptor tRNAs in genomes, their coadaptation, and potential levels of gene expression due to selection for translational efficiency [6, 7]. 

In this study, the codon usage patterns of all the available 56 sequenced retroviruses' genomes (from GenBank) containing 246 ORFs (longer than 150 bp) were analyzed. Results from this study would be useful for revealing retroviral gene composition and evolution and additionally may be useful in selecting appropriate host expression systems to improve the expression of target genes *in vivo* and *in vitro* for the design of gene delivery and expression systems for use in gene therapy and immunization.

## 2. Materials and Methods

56 completely sequenced retroviral genomes were available from NCBI GenBank (February 2010). These belonged to two major subfamilies: Orthoretrovirinae, Spumaretrovirinae and rest of the viruses were unclassified. Six viruses belong to Spumaretrovirinae, while 3 viruses were unclassified. The remaining 47 viruses belong to Orthoretrovirinae subfamily. Six genera are present within Orthoretrovirinae, namely: *Alpharetrovirus, Betaretrovirus, Deltaretrovirus*, *Epsilonretrovirus*, *Gammaretrovirus*, and *Lentivirus*. Among the 47 Orthoretrovirinae viruses, there are 7 *Alpharetroviruses*, 6 *Betaretroviruses*, 8 *Deltaretroviruses*, 2 *Epsilonretroviruses*, 14 *Gammaretroviruses,* and 10 *Lentiviruses*. 246 ORFs corresponding to all the completely sequenced genomes were available in GenBank. Only the genes with a length greater than or equal to 150 bp have been considered for further study. All these sequences together contained 135,304 of codons.

The various statistical parameters characterizing synonymous codon frequency, codon bias, base composition of whole genes, base composition at 3rd codon positions, relative gene expression levels, preferred and optimal codons, correspondence and cluster analysis on codon usage, and the associated means, standard deviations (SD), correlation coefficients, and chi-square statistics (*χ* ^2^) were computed using CodonW [8], GCUA [9], and STATISTICA 8.0 (http://www.statsoft.com/).

## 3. Results

### 3.1. Codon and Nucleotide Bias

The “Effective Number of Codons” (ENc) of a gene sequence measures the degree of bias in codon usage in the gene [10]. It ranges from 20 to 61, with values below 35 implying high bias while above 50 implying low bias. It is found that none of the retroviral genes had any strong codon bias. Around 50% of the genes had weak codon bias, implying that the rest half of the genes are moderately/randomly biased. Approximately 80% of the moderately biased genes belong to Orthoretorvirinae, 50% belong to *Lentivirus*, and 13% to *Deltaretrovirus*. *Spumaretorvirinae* and “other Orthoretrovirinae groups” contain some amount of the moderately biased genes (Table 1). Some retroviruses show more codon bias than the others. These are FIV, SFV-3, VISNA, OLV, and HIV-1 whose average ENc lies between 40 and 45. All of these are *Lentiviruses*, except SFV-3 which belongs to *Spumaretorvirinae*. The total range of ENc for all the retroviruses taken together was from 40 to 60.

Nucleotide preferences are usually an indication for the nature of mutational bias in genes or genomes. Here, in retroviruses, explicit differences are observed in nucleotide preferences. The AU content (overall A + U) of genes in single retrovirus ranged from 35% to about 60% (Table 1). AU3 (A + U content of the 3rd synonymous codon position) content in retroviruses varied over a large range, from about 20% to 75%. FIV had the highest, both AU (60%) and AU3 (70%) content. FFV, SFV-3, VISNA, OLV, and HIV-1 also had similar levels of high AU content. These viruses also had relatively higher codon bias among the whole set of retroviruses. ACMHV-2 had the lowest AU (35%) and AU3 (20%) content. Among all the retroviruses, FuSV, Y73SV, AMCV, WMSV, HTLV-4, HTLV-1, and STLV-2 had low AU (35 to 45%) and AU3 (20 to 45%) content. It was observed that AU content of the genes correlated strongly with their AU3 content (*r* = 0.91, *P* < 0.0001). AU1 and AU2 content (AU contents at first and second codon positions of genes, resp.) of retroviral genes are both about 50%. This indicates an almost equal preference for AU and GC in both the first and second codon positions. But, in general, AU12 content (AU1 + AU2) is notably less than AU3 of retroviral genes as a whole.

**Figure 1:**
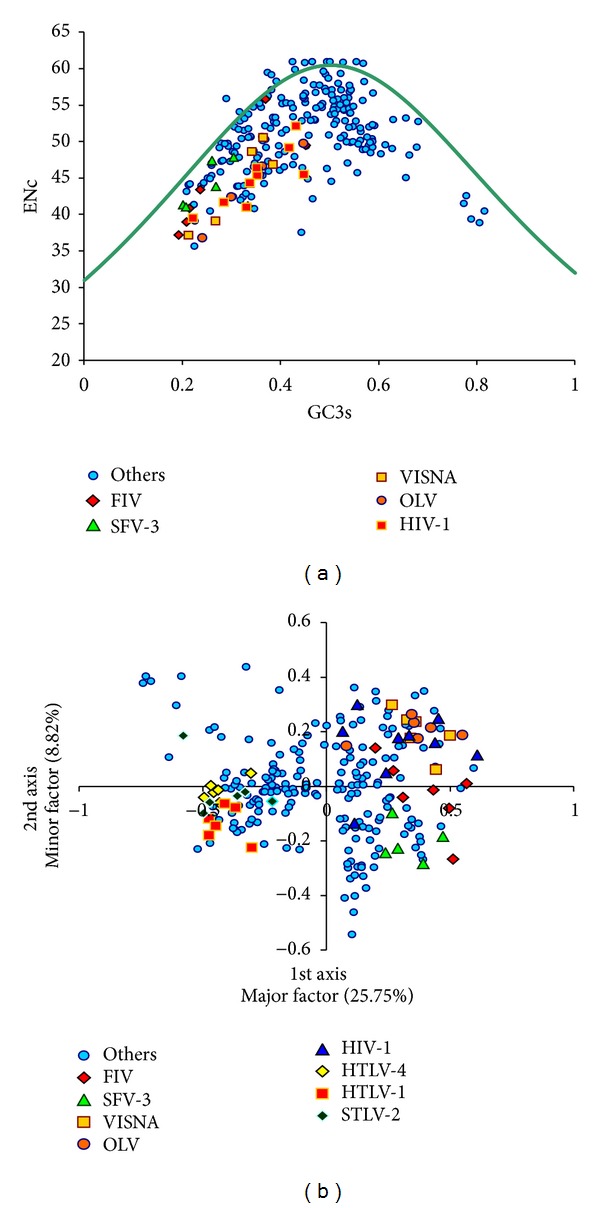
(a) ENc versus GC3 plot of all the genes. The reference viruses are in different colors. (b) The values of the first axis and the second axis of each gene in CoA. Genes from reference retroviruses are shown in different colors; genes from other viruses are plotted in blue colour.

When ENc versus AU3 content is plotted for the whole dataset, it is seen that only a small number of genes lie on the expected curve (the curve representing the variation of codon bias when determined by base composition only), while majority of the genes with low ENc values were lying well below it (Figure 1(a)). In the viruses with relatively higher codon bias, most of the genes with moderate codon bias have high AU3 (i.e., low GC) content. Furthermore, while considering the length of retroviral genes, it was found that no significant correlation existed between it and ENc of genes.

### 3.2. Preferred and Optimal Codons

Codons occurring in high frequencies in the total codon usage data of an organism are called preferred codons. Here, in retroviruses, significant differences (using *χ* ^2^ test) in overall codon usage frequencies were observed between the pairwise combinations of retroviral clades. But some pairs of viruses—*Betaretrovirus* and Spumaretrovirinae*/Epsilonretrovirus; Epsilonretrovirus and Betaretrovirus/Lentivirus *or other unclassified retroviruses; and *Gammaretrovirus* and *Deltaretrovirus* or the remaining unclassified retroviruses—were exceptions and did not show significant differences in their overall codon usage frequencies (*P* > 0.05). Four codons, GAA (Glu), AAA (Lys), AGA (Arg), and GGA (Gly), were particularly preferred to a large extent in retroviruses. Seven other codons, UUU (Phe), UUA (Leu), UAU (Tyr), CAA (Gln), AAU (Asn), GAU (Asp), and UGU (Cys), are also frequently preferred (see Supplementary Material available online at http://dx.doi.org/10.1155/2013/848123). It was also observed that closely related viruses, for example, those within the Orthoretrovirinae subfamily and those which are phylogenetically relatively closer to this group, prefer similar set of codons. It was observed especially among the viruses within genera like *Betaretrovirus, Deltaretrovirus, *Spumaretrovirinae, and *Lentivirus*. On the other hand, the *Alpharetroviruses* and *Gammaretroviruses* were somewhat of an exception with less similarity in their set of preferred codons. In general, the preferred codons almost always had A or U at their 3rd synonymous codon positions (in *Betaretrovirus, Epsilonretrovirus, Lentivirus, and *Spumaretrovirinae). But some viruses (*Alpharetrovirus, Gammaretrovirus, *and* Deltaretrovirus*) were exceptions, with high G or C content at their 3rd codon positions of preferred codons. It is also observed that the choice of preferred codons correlated with the overall genomic composition of the viruses. AU rich genomes preferred AU ending codons, and GC rich genomes preferred GC ending codons.

The codon adaptation index (CAI) is one measure that is used to estimate the extent of bias towards codons that are preferred in highly expressed genes. The CAI value ranges from 0 and 1.0 for a gene, where a higher value is likely to indicate stronger codon usage bias and a potential higher expression level. Higher CAI for a large set of genes may also indicate that selection for translation is active over that set of genes. Codons whose frequencies of usage were significantly higher in the genes with higher CAI, than that of the genes with lower CAI, are considered as the optimal codons. In this study, codon usage of retroviruses was compared (with chi-squared contingency test) between two groups of genes. One group of genes was constituted from 5% of the total number of genes, which had the maximum CAI values. The other group of genes was similarly constructed from the genes having minimum CAI. In all, 26 codons, UUU (Phe), UUA, UUG, CUA (Leu), AUA (Ile), GUA (Val), UAU (Tyr), CAU (His), CAA (Gln), AAU (Asn), AAA (Lys), GAU (Asp), GAA (Glu), UCU, UCA, AGU (Ser), CCU, CCA (Pro), ACU, ACA (Thr), GCU, GCA (Ala), UGU (Cys), AGA, AGG (Arg), and GGA (Gly), were identified as the optimal codons (*P* < 0.01) (Table 2). Almost all of these codons have an A or U at the third position. It may be noted that the previously identified preferred codons are a subset of these optimal codons. Furthermore, it was found that significant correlations exist between CAI and ENc, AU and AU3 values (*r* = 0.23, −0.32, −0.44 resp., *P* < 0.05) over the whole data set of retroviral genes. As expected if mutational bias is the main factor explaining codon usage bias in retroviruses, the frequency of preferred codons, as defined by most frequently used codons, is positively correlated with average AU composition.

### 3.3. Correspondence and Cluster Analysis

**Figure 2:**
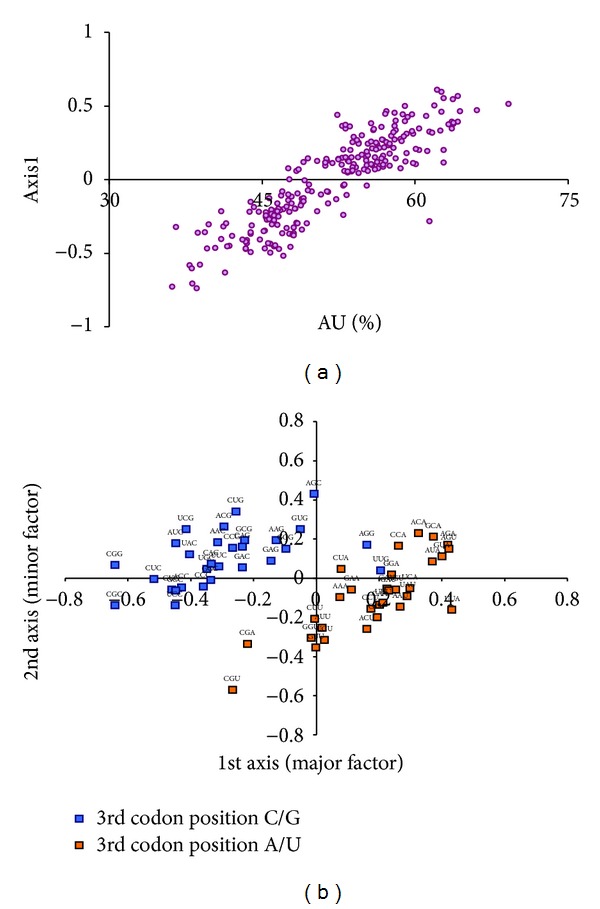
(a) Correlation between AU content of each retroviral gene and their position on the first axis of CoA. (b) The distribution of synonymous codons is shown along the first and second axes of the CoA. Codons ending with G or C are shown in blue colors, and codons ending with A or U are shown in orange colour.

Correspondence analysis (CoA) on relative synonymous codon usage (RSCU) is a method for identifying major trends/factors (as orthogonal axes) responsible for the variation in codon usage among genes represented as 59- (number of sense codons) dimensional vectors. In the correspondence analysis on codon usage of retroviral genes, the two axes which accounted for the largest amount of variations, accounted for about 25% (major axis) and 10% of the variation of the whole data set. Each of the remaining axes accounted for less than 5% of the variation. The retroviral genes were widely distributed along the length of the first major axis. Genes belonging to differently biased viruses were distinctly separated on the first major axis. The AU rich retroviruses, for example, FIV, SFV-3, VISNA, OLV, and HIV-1, were on the extreme right, while the GC rich viruses were on the other end (Figure 1(b)). It was interesting to note that *Lentiviruses* were closer to each other on the axis than to viruses from other retroviral genera. AU, AU3, and CAI correlated strongly with the first major axis (*r* = 0.89, 0.9, −0.52, resp., *P* < 0.05) (Figure 2(a)). The plot of the codons on first and second axes reveals that the A/U-ending and G/C-ending synonymous codons are also clearly separated along the first major axis (Figure 2(b)).

**Figure 3:**
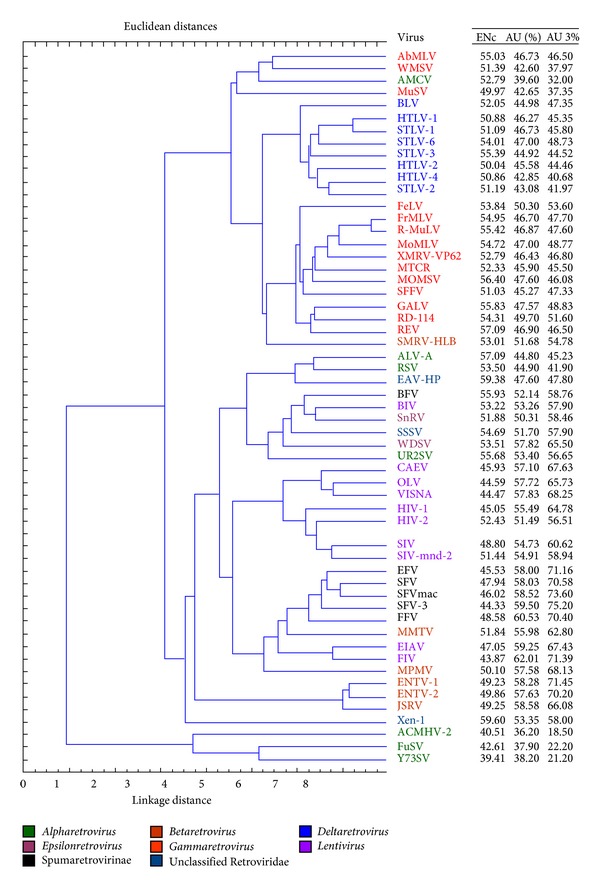
Dendogram representing the extent of divergence in relative synonymous codon usage of 56 retroviruses, using unweighed pair group average clustering, and distances are in Euclidean distance. Different clades are in different colors. To the extreme right mean ENc, mean AU% and AU3% are added from Table 1.

Cluster analysis based on codon usage reveals the grouping within and across the organisms based on the similarities and differences in their codon usage. The organisms are grouped based on a distance measure which is proportional to the similarities of the codon usage between pairs of organisms. Cluster analysis on retroviral codon usage revealed that the retroviruses are grouped into two major clusters (Figure 3), the larger cluster being further divided into two subclusters. *Alpharetroviruses* with single genes constituted the minor cluster, while all the remaining viruses constituted the major cluster. It was observed that phylogenetically closely related retroviruses are relatively closer to each other in the clusters. Some retroviruses are relatively biased, grouped together in one subcluster. Retroviruses with higher AU and AU3 form one subcluster, while those with higher GC and GC3 form the other subcluster. It is seen that *Lentiviruses* are grouped with *Betaretrovirus, *Spumaretrovirinae*, Epsilonretrovirus*, some *Alpharetrovirus,* and unclassified retroviruses, which have higher AU and AU3. Delta and Gamma retroviruses exist in the other subcluster with GC and GC3 rich viruses. This observation was in accordance with the findings from CoA.

## 4. Discussion

Retroviruses are an extremely important system for study, especially so because of its potential to adversely affect the quality of life and life-span of a large fraction of the world population especially in developing countries. These viruses are a potential threat to mankind, because of their complex biological mechanisms and evolution. This study aims to reveal the nature of some important genetic, genomic, and evolutionary features of these viruses which may be further utilized in better understanding of the retroviral system and has been designed to elucidate the general complexity and preferences of codon usage of all the retroviruses based on certain well-established parameters. Analysis of codon usage and base composition of retroviral genes documented here have revealed some useful facts. Furthermore, the results obtained through the various analyses were found to be consistent with each other, thus strongly validating the results obtained.

The large majority of the completely sequenced 56 retroviruses belonged to the Orthoretrovirinae subfamily. Within the Orthoretrovirinae, different genera contained almost equal number of viruses. Several features of retroviruses have been revealed through computation and analysis of different well-established parameters to understand their compositional and codon usage characteristics. They are RSCU, codon bias (ENc), base content, preferred and optimal codons, major factors of CoA, and grouping by cluster analysis of these viruses based on their codon usages.

It is found that retroviral genes do not possess significantly high codon bias. The genes are almost equally distributed between weak bias and moderate bias. This observation is very similar to the findings of Jenkins and Holmes, in 2003, where they had also observed moderate bias in 50 human RNA viruses [7]. However, the study by Jenkins and Holmes included only 4 human retroviruses, and in the present study a total of 56 completely sequenced retroviral sequences were analyzed, thus providing a more complete view of the total *Retroviridae* family that strongly supports the earlier observations by Jenkins and Holmes [7]. In addition, CoA was also performed in this present study that successfully discriminates between differently biased groups and added a new dimension in explaining the factors responsible for shaping the codon usage bias of the retroviruses. Large majority of the moderately biased genes belonged to the viruses from the Orthoretrovirinae subfamily. Among the total biased genes, most of the genes were from only FIV, SFV-3, VISNA, OLV, and HIV-1. There are large variations in the nucleotide composition of the retroviral genes. The AU content of the genes varied over a wide range of about 25% (range size), while the AU3 content varied over far larger range of 55% (range size). This varying pattern of AU content in retroviruses is in good agreement with the earlier study by Jenkins and Holmes [7]. Four of the human retroviruses reported by Jenkins and Holmes and in this present study also followed similar base usage pattern [7]. Viruses, which exhibited higher codon usage bias (mentioned earlier), also possessed higher AU content, both at the third synonymous codon position and in overall gene composition. FIV had the highest AU and AU3 content over other retroviruses, while ACMHV-2 had the lowest. Though AU3 content increased with overall AU content, there was no specific nucleotide bias in the major fraction of first and second codon positions of retroviruses. AU content of the first and second codon positions combined is relatively higher in viruses which have relatively higher codon bias and higher AU & AU3 content. There are some notable variations of base content of genes and codon bias even within retroviral genomes. Thus, heterogeneity of compositional bias exists both within and across retroviral genomes. Additionally, it is also clear from data that codon usage and base composition are virus-specific to a considerable extent. Majority of the genes were below the expected curve of codon bias when plotted against base composition of the third codon position (Figure 1(a)). This signifies that additional factors other than base composition might also have influenced retroviral codon usage.

Some retroviruses were significantly similar in their overall codon usage, while majority was not. Four preferred codons were identified, all of which were subset of the set of 26 optimal codons separately identified. It was observed that phylogenetically closer retroviruses possess relatively similar codon usage and almost the same sets of preferred and optimal codons having A or U in their synonymous positions. But *Alpharetrovirus, Gammaretrovirus, *and* Deltaretrovirus* were exceptions with relatively higher G or C in their 3rd codon positions. Compositions of the optimal codons were correlated with the average genetic base composition of these viruses. In correspondence analysis, the two dominant axes accounted for about one-third of the total variation of codon usage in retroviruses. It was seen that these axes successfully differentiated the genes based on codon bias, base content, and codon composition. Correlation between CAI, as a measure of relative expression and first major axis, indicates that translational selection seemingly has a role in retroviruses. This observation implicates that once the viral genome enters into host translationary mechanism, then the biased genes (indicated by ENC and 1st major axis) having higher relative expression potential (higher CAI value) that matches the host can translate faster, thus achieving higher fitness for the virus [11]. These results and correlations between CAI, codon bias, and base composition indicate that genes with relatively higher codon bias are primarily composed of mostly optimal codons. Cluster analysis also validated that evolutionary-related retroviruses have similar codon usage, and those which are distant have distinctly different codon usage. 

In the light of the general fact that selective constraints are greater in the first two positions of codons, whereas mutational bias is greater in the third position, all the observations indicate that codon bias in retroviruses in general is strongly dependent on base composition and mutational bias. This observation is also supported by earlier studies where it has been shown that main factor explaining codon usage in viruses is mutation bias [7, 12–14] and generally AT mutation bias in RNA viruses [15, 16]. Selection for overall efficient expression for genes is probably an important factor affecting codon usage in these systems (as indicated by correlation between CAI and the first major axis). Groupings observed from cluster analysis and the conservation of preferred and optimal codons and similar base usage in phylogenetically close retroviruses indicate that codon usage and nucleotide composition might have evolved through a concerted process in these viral systems. There is a certain possibility that overall all AU richness of the retroviruses, being host dependent viruses, might have evolved due to differential cost and exploit the availability of relevant metabolites in the host cell [17]. This is also supported by the fact that small genome viruses, like retroviruses, are more AU prone than in contrast to large DNA viruses which are mostly GC rich [18]. Evidence of translational selection on codon usage bias in the viruses has been found on a subset of genes only, those for which selection efficiency or accuracy is possibly the most important for the survival of the viruses based on their improved expression [19, 20]. This fact is also supported by earlier studies, where correlation between viral codon usage bias and host codon usage bias implicates better exploitation of the host translationary mechanism in cooperation with the host translational bias [11, 20].

There is a good possibility that compositional bias detected in retroviruses in this study is the result of a directional mutational pressure imposed by one of the two enzymes that copies the retroviral genome, that is, retrovirus-specific reverse-transcriptase (RT) enzyme, which converts the viral RNA into DNA. It is a distinct possibility that the absence of any strong codon bias in retroviruses might be due to the combined effect of missincorporations by the error-prone RT polymerase enzyme (mentioned above) and another class of enzyme, cytidine deaminases such as enzymes of APOBEC3 superfamily [21, 22] and lack of strong selection on codons in retroviral genes. There is indeed some experimental evidence that the HIV-1 RT enzyme is responsible for accumulation of A nucleotides in viral plus-strand genome. These enzymes are also supposed to be responsible for hypermutation of retroviruses, such as HIV-1 and SIV [22, 23]. Relatively error-prone RT polymerase enzyme and enzymes like APOBEC3 cytidine deaminases preferentially incorporate G to U mismatches during minus strand cDNA synthesis [21, 23] and eventually further induces G to A mutations in the viral RNA genome [24]. It is possible that other retroviruses also have evolved under the influence of similar enzymes, inducing hypermutation in those viruses. Relations observed in this study between distinct AU preference and codon usage bias in retroviruses may be due to this general propensity of G to U and further to A mutation. In fact, absence of relation between gene length and codon usage, implying the absence of strong selection for translational *accuracy*, specifically, in these viruses, might be due to the effect of missincorporations by the error-prone, RT polymerase itself [21] and cytidine deaminases [23]. Weak codon bias observed is possibly the result of these high mutation rates in retroviruses. This might be advantageous for maintaining high mutation rates in these viruses. Such rapid mutation, for example, in HIV-1, leads to an accumulation of diversity of its gene sequences. By diversifying, the viruses are probably able to escape host immune detection. But this phenomenon must be occurring at the expense of purifying effect of the general selection forces [25, 26]. Furthermore, the weak codon bias in retroviruses might also be contributing towards decreasing host immune response during retroviral infection in the host by allowing the relatively lower expression of viral “env” to be suppressed in order to minimize antigenic profile of these viruses [27]. These critical processes may have shaped retroviral genes over time to become a very successful immunoinvading system. There are some lines of evidence that suggest that retroviruses, such as HIV-1, are subject to a positive selection pressure imposed by the immune system [28]. Additionally, previous studies indicate that retroviral gene expression is controlled by multiple complex regulatory mechanisms [29]. For example, HIV structural proteins are expressed from unspliced 9 kb (gag/pol) and partially spliced 4 kb (env) transcripts that are unstable and can efficiently be exported from the nucleus in absence of HIV regulatory protein Rev. The lack of nuclear stability and export in absence of Rev is partly due to the presence of defined inhibitory sequences (known as INS, IN, and CRS) within structural genes themselves. In this context, the low GC content of HIV RNA also contributes to nuclear instability, even in absence of defined inhibitory sequences [30]. All these facts may help to put in context the compositional patterns and codon usage bias in majority of retroviruses.

Observations from comparative analysis of codon usage bias reveal lack of strong translational selection in considerable number of retroviruses and this could be a problem of using retroviruses as expression vectors for gene therapy and immunization. Instead, use of the retroviruses with AU rich nucleotide composition is recommended, utilizing optimal set of codons. Information on optimal codons obtained from this study is expected to be useful for codon optimization especially for designing retroviral vectors with higher translational efficiency and production of simple and safe retroviral vectors for gene therapy and immunization.

## 5. Conclusion

Overall, the results point towards the fact that mutational bias is a dominant factor, relative to translational selection, in shaping codon usage of retroviruses. In these viruses, where codon usage bias is not strong, it is primarily determined by base composition, that is, AU (or GC) content of the genes, while selection for efficient expression for genes is probably another important factor affecting their codon usage. The intricate character of codon usage of these viral systems is probably maintained by incorporations of errors during molecular processing of the retroviral genomes, to help avoid strong immune response from the infected host but yet strike a balance with adequate execution of basic life cycle mechanisms of these viruses. In spite of inter- and intra-genomic differences of base and codon usage, it is possible that the extant retroviruses, in general, have emerged through a complex but concerted process of evolution.

## Supplementary Material

Supplementary data table lists the RSCU and codon usage values of different retrovirus groups. The list shows that some of the codons are more frequently observed than others, while coding for the same amino acid. This table provides insight about the preferred codons.

## Figures and Tables

**Table 1:** Characteristics and codon usage pattern and AU distribution of retroviral genomes (shown in clades).

Virus*	Abbrev. names	Accn. number	Mean ENc	SD	Mean AU %	SD	Mean AU3 %	SD
Orthoretrovirinae								
* Alpharetrovirus *								
* Avian carcinoma virus *	ACMHV-2	NC_001402	40.51	0.00	36.20	0.00	18.50	0.00
* Avian leukosis virus*—*RSA *	ALV-A	NC_001408	57.09	3.32	44.80	7.35	45.23	9.32
* Avian myelocytomatosis virus *	AMCV	NC_001866	52.79	0.00	39.60	0.00	32.00	0.00
* Fujinami sarcoma virus *	FuSV	NC_001403	42.61	0.00	37.90	0.00	22.20	0.00
* Rous sarcoma virus *	RSV	NC_001407	53.50	9.93	44.90	5.28	41.90	15.25
* UR2 sarcoma virus *	UR2SV	NC_001618	55.68	0.59	53.40	0.00	56.65	1.77
* Y73 sarcoma virus *	Y73SV	NC_008094	39.41	0.00	38.20	0.00	21.20	0.00
* Betaretrovirus *								
* Enzootic nasal tumour virus of goats *	ENTV-2	NC_004994	49.86	1.36	57.63	2.33	70.20	1.53
* Jaagsiekte sheep retrovirus *	JSRV	NC_001494	49.25	4.26	58.58	2.38	66.08	9.57
* Mason-Pfizer monkey virus *	MPMV	NC_001550	50.10	1.31	57.58	1.27	68.13	1.60
* Mouse mammary tumor virus *	MMTV	NC_001503	51.84	2.16	55.98	0.62	62.80	2.42
* Ovine enzootic nasal tumour virus *	ENTV-1	NC_007015	49.23	2.80	58.28	2.72	71.45	2.87
* Squirrel monkey retrovirus*—*HLB *	SMRV-HLB	NC_001514	53.01	4.61	51.68	2.01	54.78	3.39
* Deltaretrovirus *								
* Bovine leukemia virus *	BLV	NC_001414	52.05	2.54	44.98	3.49	47.35	4.83
* Primate T-lymphotropic virus 1 *								
* Human T-lymphotropic virus 1 *	HTLV-1	NC_001436	50.88	1.62	46.27	2.05	45.35	2.82
* Simian T-lymphotropic virus 1 *	STLV-1	NC_000858	51.09	2.06	46.73	2.29	45.80	3.45
* Primate T-lymphotropic virus 2 *								
* Human T-lymphotropic virus 2 *	HTLV-2	NC_001488	50.04	1.86	45.58	3.31	44.46	3.11
* Simian T-lymphotropic virus 2 *	STLV-2	NC_001815	51.19	4.24	43.08	3.82	41.97	5.16
* Human T-lymphotropic virus 4 *	HTLV-4	NC_011800	50.86	2.71	42.85	2.62	40.68	2.23
* Simian T-cell lymphotropic virus 6 *	STLV-6	NC_011546	54.01	3.83	47.00	2.78	48.73	4.68
* Simian T-lymphotropic virus 3 *	STLV-3	NC_003323	55.39	3.72	44.92	2.73	44.52	2.31
* Epsilonretrovirus *								
* Snakehead retrovirus *	SnRV	NC_001724	51.88	7.91	50.31	4.73	58.46	4.80
* Walleye dermal sarcoma virus *	WDSV	NC_001867	53.51	2.45	57.82	3.39	65.50	3.56
* Gammaretrovirus *								
* Abelson murine leukemia virus *	AbMLV	NC_001499	55.03	6.45	46.73	8.81	46.50	11.23
* Feline leukemia virus *	FeLV	NC_001940	53.84	4.16	50.30	1.70	53.60	0.99
* Murine leukemia virus *								
* Friend murine leukemia virus *	FrMLV	NC_001362	54.95	1.03	46.70	1.41	47.70	1.82
* Moloney murine leukemia virus *	MoMLV	NC_001501	54.72	0.10	47.00	1.84	48.77	2.80
* Murine type C retrovirus *	MTCR	NC_001702	52.33	3.00	45.90	0.99	45.50	2.44
* Rauscher murine leukemia virus *	R-MuLV	NC_001819	55.42	0.62	46.87	1.40	47.60	1.32
* Gibbon ape leukemia virus *	GALV	NC_001885	55.83	1.34	47.57	1.16	48.83	0.67
* Moloney murine sarcoma virus *	MOMSV	NC_001502	56.40	3.24	47.60	6.26	46.08	8.42
* Murine osteosarcoma virus *	MuSV	NC_001506	49.97	0.98	42.65	1.77	37.35	7.14
* RD114 retrovirus *	RD-114	NC_009889	54.31	1.93	49.70	3.68	51.60	3.25
* Reticuloendotheliosis virus *	REV	NC_006934	57.09	0.20	46.90	0.80	46.50	1.44
* Spleen focus-forming virus *	SFFV	NC_001500	51.03	5.85	45.27	2.28	47.33	3.37
* Woolly monkey sarcoma virus *	WMSV	NC_009424	51.39	8.65	42.60	4.04	37.97	13.23
* Xenotropic MuLV-related virus VP62 *	XMRV-VP62	NC_007815	52.79	2.41	46.43	1.29	46.80	2.18
*Lentivirus *								
*Bovine immunodeficiency virus *	BIV	NC_001413	53.22	4.43	53.26	3.60	57.90	5.35
*Caprine arthritis-encephalitis virus *	CAEV	NC_001463	45.93	6.96	57.10	3.45	67.63	4.46
*Equine infectious anemia virus *	EIAV	NC_001450	47.05	7.93	59.25	4.56	67.43	1.18
*Feline immunodeficiency virus *	FIV	NC_001482	43.87	6.58	62.01	3.83	71.39	9.91
*Human immunodeficiency virus 1 *	HIV-1	NC_001802	45.05	4.01	55.49	4.66	64.78	7.24
*Human immunodeficiency virus 2 *	HIV-2	NC_001722	52.43	5.73	51.49	3.02	56.51	4.77
*Ovine lentivirus *	OLV	NC_001511	44.59	4.47	57.72	3.78	65.73	6.90
*Simian immunodeficiency virus *								
*Simian immunodeficiency virus *	SIV	NC_001549	48.80	4.12	54.73	3.32	60.62	7.82
*Simian immunodeficiency virus SIV-mnd 2 *	SIV-mnd-2	NC_004455	51.44	5.83	54.91	2.63	58.94	6.43
*Visna/Maedi virus *	VISNA	NC_001452	44.47	5.34	57.83	2.70	68.25	6.51
Spumaretrovirinae								
*Bovine foamy virus *	BFV	NC_001831	55.93	2.36	52.14	3.66	58.76	5.22
*Equine foamy virus *	EFV	NC_002201	45.53	2.69	58.00	4.83	71.16	5.47
*Feline foamy virus *	FFV	NC_001871	48.58	3.78	60.53	2.95	70.40	3.59
*Macaque simian foamy virus *	SFVmac	NC_010819	46.02	3.01	58.52	4.92	73.60	5.31
*Simian foamy virus *	SFV	NC_001364	47.94	3.91	58.03	4.38	70.58	8.48
*Simian foamy virus 3 *	SFV-3	NC_010820	44.33	3.23	59.50	5.31	75.20	4.35
Unclassified retroviruses								
*Atlantic salmon swim bladder sarcoma virus *	SSSV	NC_007654	54.69	2.28	51.70	3.54	57.90	6.65
*Avian endogenous retrovirus EAV-HP *	EAV-HP	NC_005947	59.38	0.00	47.60	0.00	47.80	0.00
*Xenopus laevis endogenous retrovirus Xen1 *	Xen-1	NC_010955	59.60	1.80	53.35	2.76	58.00	4.11

*Viruses are shown in their respective genera.

**Table 2:** Translational optimal codons.

Amino acid	Codon^#^	High		Low	
		RSCU	Number	RSCU	Number
Phe	UUU*	1.64	184	0.7	61
	UUC	0.36	40	1.3	113

Leu	UUA*	2.67	323	0.34	35
	UUG*	1.21	146	0.5	51
	CUU	0.47	57	0.63	65
	CUC	0.29	35	1.83	189
	CUA*	0.94	114	0.53	55
	CUG	0.41	50	2.17	223

Ile	AUU	0.85	193	0.77	55
	AUC	0.29	66	1.72	122
	AUA*	1.86	425	0.51	36

Val	GUU	0.65	80	0.63	44
	GUC	0.36	44	1.38	96
	GUA*	2.17	267	0.32	22
	GUG	0.82	101	1.68	117

Tyr	UAU*	1.74	270	0.44	34
	UAC	0.26	41	1.56	121

His	CAU*	1.5	144	0.64	63
	CAC	0.5	48	1.36	134

Gln	CAA*	1.49	383	0.47	71
	CAG	0.51	131	1.53	228

Asn	AAU*	1.67	317	0.54	38
	AAC	0.33	62	1.46	104

Lys	AAA*	1.38	481	0.68	93
	AAG	0.62	218	1.32	182

Asp	GAU*	1.51	259	0.53	54
	GAC	0.49	85	1.47	149

Glu	GAA*	1.5	474	0.61	99
	GAG	0.5	159	1.39	228

Ser	UCU*	1.18	77	0.76	49
	UCC	0.56	37	2.1	136
	UCA*	1.88	123	0.51	33
	UCG	0.2	13	0.71	46
	AGU*	1.53	100	0.37	24
	AGC	0.66	43	1.56	101

Pro	CCU*	1.47	162	0.68	81
	CCC	0.52	57	2.04	243
	CCA*	1.78	196	0.75	89
	CCG	0.24	26	0.54	64

Thr	ACU*	1.29	156	0.67	57
	ACC	0.42	51	2.1	178
	ACA*	2.09	252	0.67	57
	ACG	0.2	24	0.55	47

Ala	GCU*	1.09	130	0.7	69
	GCC	0.58	69	2.11	207
	GCA*	2.07	246	0.67	66
	GCG	0.26	31	0.51	50

Cys	UGU*	1.69	133	0.5	27
	UGC	0.31	24	1.5	81

Arg	CGU	0.03	2	0.53	26
	CGC	0.09	6	1.64	81
	CGA	0.42	28	0.65	32
	CGG	0.12	8	2.03	100
	AGA*	3.71	250	0.45	22
	AGG*	1.63	110	0.71	35

Gly	GGU	0.49	73	0.42	37
	GGC	0.32	48	1.52	134
	GGA*	2.22	333	0.74	65
	GGG	0.97	145	1.33	117

^#^W, M, and stop codons are excluded. Those codons are significantly higher in highly expressed genes.
