# Supplementary material for: Complex Codon Usage Pattern and Compositional Features of Retroviruses
Source: Comput Math Methods Med. 2013 Oct 31;2013:848123. doi: 10.1155/2013/848123 (PMC3833384; doi:10.1155/2013/848123)
Supplement: Supplementary file 1 — Supplementary data table lists the RSCU and codon usage values of different retrovirus groups. The list shows that some of the codons are more frequently observed than others, while coding for the same amino acid. This table provides insight about the preferred codons. [file 848123.f1.doc]

**Supplementary data table 1**

| **RSCU and Codon usage in different retrovirus groups** | | | | | | | | | | | | | | | | | | | | | | | | | |
| --- | --- | --- | --- | --- | --- | --- | --- | --- | --- | --- | --- | --- | --- | --- | --- | --- | --- | --- | --- | --- | --- | --- | --- | --- | --- |
| **Orthoretrovirinae Spumaretrovirinae Unclassified**  **_________________________________________________________________________________________________ Retroviridae** | | | | | | | | | | | | | | | | | | | | | | | | | |
| Alpharetrovirus Betaretrovirus Deltaretrovirus Epsilonretrovirus Gamaretrovirus Lentivirus | | | | | | | | | | | | | | | | | | | | | | | | | |
| **aa Codon**^#^ **No RSCU % No RSCU % No RSCU % No RSCU % No RSCU % No RSCU % No RSCU % No RSCU %** | | | | | | | | | | | | | | | | | | | | | | | | | |
| **F** | **UUU** | 104 | 0.97 | 48.60 | **472** | **1.45** | **72.50** | 287 | 0.85 | 42.64 | **149** | **1.27** | **63.40** | 327 | 0.96 | 47.81 | **569** | **1.31** | **65.63** | **386** | **1.48** | **74.23** | **68** | **1.27** | **63.55** |
|  | UUC | **110** | **1.03** | **51.40** | 179 | 0.55 | 27.50 | **386** | **1.15** | **57.36** | 86 | 0.73 | 36.60 | **357** | **1.04** | **52.19** | 298 | 0.69 | 34.37 | 134 | 0.52 | 25.77 | 39 | 0.73 | 36.45 |
|  |  |  |  |  |  |  |  |  |  |  |  |  |  |  |  |  |  |  |  |  |  |  |  |  |  |
| **L** | **UUA** | 119 | 0.77 | 12.75 | **571** | **1.89** | **31.46** | 325 | 0.71 | 11.90 | **191** | **1.51** | **25.16** | 396 | 0.81 | 13.56 | **833** | **1.77** | **29.58** | **574** | **1.75** | **29.12** | 46 | 0.69 | 11.47 |
|  | UUG | 164 | 1.05 | 17.58 | 272 | 0.9 | 14.99 | 206 | 0.45 | 7.55 | 141 | 1.11 | 18.58 | 327 | 0.67 | 11.19 | 480 | 1.02 | 17.05 | 380 | 1.16 | 19.28 | 82 | 1.23 | 20.45 |
|  | CUU | 112 | 0.72 | 12.00 | 376 | 1.24 | 20.72 | 485 | 1.07 | 17.77 | 96 | 0.76 | 12.65 | 337 | 0.69 | 11.54 | 264 | 0.56 | 9.38 | 358 | 1.09 | 18.16 | 67 | 1 | 16.71 |
|  | CUC | 164 | 1.05 | 17.58 | 205 | 0.68 | 11.29 | **836** | **1.84** | **30.62** | 68 | 0.54 | 8.96 | 655 | 1.35 | 22.42 | 297 | 0.63 | 10.55 | 154 | 0.47 | 7.81 | 51 | 0.76 | 12.72 |
|  | CUA | 64 | 0.41 | 6.86 | 208 | 0.69 | 11.46 | 509 | 1.12 | 18.64 | 155 | 1.23 | 20.42 | 535 | 1.1 | 18.32 | 528 | 1.12 | 18.75 | 275 | 0.84 | 13.95 | 64 | 0.96 | 15.96 |
|  | CUG | **310** | **1.99** | **33.23** | 183 | 0.6 | 10.08 | 369 | 0.81 | 13.52 | 108 | 0.85 | 14.23 | **671** | **1.38** | **22.97** | 414 | 0.88 | 14.70 | 230 | 0.7 | 11.67 | **91** | **1.36** | **22.69** |
|  |  |  |  |  |  |  |  |  |  |  |  |  |  |  |  |  |  |  |  |  |  |  |  |  |  |
| **Y** | **UAU** | 84 | 0.91 | 45.65 | **400** | **1.49** | **74.63** | 232 | 0.82 | 40.77 | **130** | **1.13** | **56.52** | 338 | 0.98 | 48.84 | **742** | **1.35** | **67.52** | **640** | **1.64** | **82.16** | **58** | **1.01** | **50.43** |
|  | UAC | **100** | **1.09** | **54.35** | 136 | 0.51 | 25.37 | **337** | **1.18** | **59.23** | 100 | 0.87 | 43.48 | **354** | **1.02** | **51.16** | 357 | 0.65 | 32.48 | 139 | 0.36 | 17.84 | 57 | 0.99 | 49.57 |
|  |  |  |  |  |  |  |  |  |  |  |  |  |  |  |  |  |  |  |  |  |  |  |  |  |  |
| **H** | CAU | 75 | 0.77 | 18.25 | **329** | **1.37** | **68.68** | 360 | 0.92 | 45.92 | **146** | **1.18** | **59.11** | 256 | 0.87 | 43.61 | **492** | **1.27** | **63.73** | **403** | **1.52** | **75.75** | 52 | 0.9 | 44.83 |
|  | CAC | **119** | **1.23** | **28.95** | 150 | 0.63 | 31.32 | **424** | **1.08** | **54.08** | 101 | 0.82 | 40.89 | **331** | **1.13** | **56.39** | 280 | 0.73 | 36.27 | 129 | 0.48 | 24.25 | **64** | **1.1** | **55.17** |
|  |  |  |  |  |  |  |  |  |  |  |  |  |  |  |  |  |  |  |  |  |  |  |  |  |  |
| **Q** | **CAA** | 111 | 0.54 | 27.01 | **708** | **1.34** | **67.11** | **709** | **1.05** | **52.44** | **319** | **1.19** | **59.29** | 536 | 0.8 | 40.24 | **1119** | **1.12** | **55.81** | **822** | **1.39** | **69.31** | 101 | 0.98 | 49.03 |
|  | CAG | **300** | **1.46** | **72.99** | 347 | 0.66 | 32.89 | 643 | 0.95 | 47.56 | 219 | 0.81 | 40.71 | **796** | **1.2** | **59.76** | 886 | 0.88 | 44.19 | 364 | 0.61 | 30.69 | **105** | **1.02** | **50.97** |
|  |  |  |  |  |  |  |  |  |  |  |  |  |  |  |  |  |  |  |  |  |  |  |  |  |  |
| **I** | AUU | 137 | 1.06 | 35.40 | **494** | **1.47** | **48.86** | 329 | 0.97 | 32.32 | 136 | 0.84 | 27.93 | 279 | 0.9 | 30.03 | 567 | 0.77 | 25.67 | **546** | **1.32** | **43.93** | 60 | 1 | 33.33 |
|  | AUC | **162** | **1.26** | **41.86** | 191 | 0.57 | 18.89 | **443** | **1.31** | **43.52** | 89 | 0.55 | 18.28 | **393** | **1.27** | **42.30** | 422 | 0.57 | 19.10 | 155 | 0.37 | 12.47 | 49 | 0.82 | 27.22 |
|  | AUA | 88 | 0.68 | 22.74 | 326 | 0.97 | 32.25 | 246 | 0.72 | 24.17 | **262** | **1.61** | **53.80** | 257 | 0.83 | 27.66 | **1220** | **1.66** | **55.23** | 542 | 1.31 | 43.60 | **71** | **1.18** | **39.44** |
|  |  |  |  |  |  |  |  |  |  |  |  |  |  |  |  |  |  |  |  |  |  |  |  |  |  |
| **N** | **AAU** | 81 | 0.7 | 35.22 | **510** | **1.35** | **67.73** | 328 | 0.88 | 43.79 | **175** | **1.01** | **50.72** | 332 | 0.88 | 43.92 | **1093** | **1.35** | **67.72** | **753** | **1.54** | **76.92** | **79** | **1.02** | **50.97** |
|  | AAC | **149** | **1.3** | **64.78** | 243 | 0.65 | 32.27 | **421** | **1.12** | **56.21** | 170 | 0.99 | 49.28 | **424** | **1.12** | **56.08** | 521 | 0.65 | 32.28 | 226 | 0.46 | 23.08 | 76 | 0.98 | 49.03 |
|  |  |  |  |  |  |  |  |  |  |  |  |  |  |  |  |  |  |  |  |  |  |  |  |  |  |
| **K** | **AAA** | 171 | 0.75 | 37.34 | **760** | **1.39** | **69.47** | **539** | **1.34** | **67.12** | **397** | **1.46** | **73.25** | **705** | **1** | **50.25** | **1542** | **1.22** | **60.97** | **748** | **1.28** | **63.93** | 86 | 0.92 | 46.24 |
|  | AAG | **287** | **1.25** | **62.66** | 334 | 0.61 | 30.53 | 264 | 0.66 | 32.88 | 145 | 0.54 | 26.75 | 698 | 1 | 49.75 | 987 | 0.78 | 39.03 | 422 | 0.72 | 36.07 | **100** | **1.08** | **53.76** |
|  |  |  |  |  |  |  |  |  |  |  |  |  |  |  |  |  |  |  |  |  |  |  |  |  |  |
| **V** | GUU | 123 | 0.89 | 22.20 | **356** | **1.52** | **37.95** | 204 | 0.9 | 22.54 | 117 | 0.99 | 24.84 | 272 | 0.73 | 18.24 | 253 | 0.52 | 12.91 | 344 | 1.2 | 30.12 | 60 | 0.93 | 23.35 |
|  | GUC | 155 | 1.12 | 27.98 | 159 | 0.68 | 16.95 | **367** | **1.62** | **40.55** | 73 | 0.62 | 15.50 | **437** | **1.17** | **29.31** | 248 | 0.51 | 12.66 | 166 | 0.58 | 14.54 | 61 | 0.95 | 23.74 |
|  | GUA | 71 | 0.51 | 12.82 | 246 | 1.05 | 26.23 | 173 | 0.76 | 19.12 | **180** | **1.53** | **38.22** | 366 | 0.98 | 24.55 | **961** | **1.96** | **49.06** | **387** | **1.36** | **33.89** | 54 | 0.84 | 21.01 |
|  | GUG | **205** | **1.48** | **37.00** | 177 | 0.75 | 18.87 | 161 | 0.71 | 17.79 | 101 | 0.86 | 21.44 | 416 | 1.12 | 27.90 | 497 | 1.01 | 25.37 | 245 | 0.86 | 21.45 | **82** | **1.28** | **31.91** |
|  |  |  |  |  |  |  |  |  |  |  |  |  |  |  |  |  |  |  |  |  |  |  |  |  |  |
| **D** | **GAU** | 155 | 0.84 | 42.23 | **587** | **1.32** | **65.81** | 276 | 0.75 | 37.65 | **209** | **1.09** | **54.29** | 536 | 0.86 | 43.05 | **788** | **1.19** | **59.43** | **655** | **1.37** | **68.44** | **102** | **1.03** | **51.52** |
|  | GAC | **212** | **1.16** | **57.77** | 305 | 0.68 | 34.19 | **457** | **1.25** | **62.35** | 176 | 0.91 | 45.71 | **709** | **1.14** | **56.95** | 538 | 0.81 | 40.57 | 302 | 0.63 | 31.56 | 96 | 0.97 | 48.48 |
|  |  |  |  |  |  |  |  |  |  |  |  |  |  |  |  |  |  |  |  |  |  |  |  |  |  |
| **E** | **GAA** | 209 | 0.7 | 35.19 | **518** | **1.28** | **63.87** | **410** | **1.26** | **63.17** | **332** | **1.33** | **66.40** | **762** | **1.04** | **52.08** | **1645** | **1.31** | **65.62** | **840** | **1.47** | **73.75** | **115** | **1.06** | **52.75** |
|  | GAG | **385** | **1.3** | **64.81** | 293 | 0.72 | 36.13 | 239 | 0.74 | 36.83 | 168 | 0.67 | 33.60 | 701 | 0.96 | 47.92 | 862 | 0.69 | 34.38 | 299 | 0.53 | 26.25 | 103 | 0.94 | 47.25 |
|  |  |  |  |  |  |  |  |  |  |  |  |  |  |  |  |  |  |  |  |  |  |  |  |  |  |
| **S** | UCU | 105 | 0.98 | 16.33 | **368** | **1.86** | **30.98** | 299 | 1.04 | 17.30 | 95 | 1.21 | 20.21 | 403 | 1.49 | 24.78 | 241 | 0.85 | 14.18 | **374** | **1.75** | **29.22** | 56 | 1.09 | 18.18 |
|  | UCC | **158** | **1.47** | **24.57** | 223 | 1.13 | 18.77 | **699** | **2.43** | **40.45** | 73 | 0.93 | 15.53 | **481** | **1.77** | **29.58** | 221 | 0.78 | 13.00 | 200 | 0.94 | 15.63 | 56 | 1.09 | 18.18 |
|  | UCA | 73 | 0.68 | 11.35 | 223 | 1.13 | 18.77 | 251 | 0.87 | 14.53 | **115** | **1.47** | **24.47** | 231 | 0.85 | 14.21 | **438** | **1.55** | **25.76** | 266 | 1.25 | 20.78 | **77** | **1.5** | **25.00** |
|  | UCG | 84 | 0.78 | 13.06 | 63 | 0.32 | 5.30 | 103 | 0.36 | 5.96 | 25 | 0.32 | 5.32 | 117 | 0.43 | 7.20 | 68 | 0.24 | 4.00 | 39 | 0.18 | 3.05 | 32 | 0.62 | 10.39 |
|  | AGU | 79 | 0.74 | 12.29 | 186 | 0.94 | 15.66 | 119 | 0.41 | 6.89 | 88 | 1.12 | 18.72 | 167 | 0.62 | 10.27 | 387 | 1.37 | 22.76 | 262 | 1.23 | 20.47 | 44 | 0.86 | 14.29 |
|  | AGC | 144 | 1.34 | 22.40 | 125 | 0.63 | 10.52 | 257 | 0.89 | 14.87 | 74 | 0.94 | 15.74 | 227 | 0.84 | 13.96 | 345 | 1.22 | 20.29 | 139 | 0.65 | 10.86 | 43 | 0.84 | 13.96 |
|  |  |  |  |  |  |  |  |  |  |  |  |  |  |  |  |  |  |  |  |  |  |  |  |  |  |
| **C** | **UGU** | 72 | 0.79 | 39.34 | **193** | **1.2** | **59.75** | 171 | 0.68 | 34.13 | **97** | **1.25** | **62.58** | 210 | 0.92 | 46.15 | **517** | **1.31** | **65.53** | **247** | **1.46** | **72.86** | **73** | **1.25** | **62.39** |
|  | UGC | **111** | **1.21** | **60.66** | 130 | 0.8 | 40.25 | **330** | **1.32** | **65.87** | 58 | 0.75 | 37.42 | **245** | **1.08** | **53.85** | 272 | 0.69 | 34.47 | 92 | 0.54 | 27.14 | 44 | 0.75 | 37.61 |
|  |  |  |  |  |  |  |  |  |  |  |  |  |  |  |  |  |  |  |  |  |  |  |  |  |  |
| **P** | CCU | 147 | 0.94 | 23.48 | **554** | **1.7** | **42.58** | 610 | 0.97 | 24.16 | **190** | **1.35** | **33.63** | 687 | 1.15 | 28.74 | 464 | 1.06 | 26.44 | **638** | **1.72** | **42.91** | 77 | 0.99 | 24.76 |
|  | CCC | **219** | **1.4** | **34.98** | 341 | 1.05 | 26.21 | **1105** | **1.75** | **43.76** | 137 | 0.97 | 24.25 | **901** | **1.51** | **37.70** | 321 | 0.73 | 18.29 | 246 | 0.66 | 16.54 | 79 | 1.02 | 25.40 |
|  | CCA | 120 | 0.77 | 19.17 | 298 | 0.92 | 22.91 | 569 | 0.9 | 22.53 | 189 | 1.34 | 33.45 | 491 | 0.82 | 20.54 | **820** | **1.87** | **46.72** | 525 | 1.41 | 35.31 | **103** | **1.32** | **33.12** |
|  | CCG | 140 | 0.89 | 22.36 | 108 | 0.33 | 8.30 | 241 | 0.38 | 9.54 | 49 | 0.35 | 8.67 | 311 | 0.52 | 13.01 | 150 | 0.34 | 8.55 | 78 | 0.21 | 5.25 | 52 | 0.67 | 16.72 |
|  |  |  |  |  |  |  |  |  |  |  |  |  |  |  |  |  |  |  |  |  |  |  |  |  |  |
| **R** | CGU | 48 | 0.54 | 8.97 | 121 | 0.97 | 16.22 | 106 | 0.63 | 10.55 | 28 | 0.39 | 6.54 | 111 | 0.4 | 6.62 | 36 | 0.11 | 1.75 | 73 | 0.42 | 7.06 | 25 | 0.62 | 10.29 |
|  | CGC | 86 | 0.96 | 16.07 | 81 | 0.65 | 10.86 | **223** | **1.33** | **22.19** | 21 | 0.29 | 4.91 | 243 | 0.87 | 14.48 | 74 | 0.22 | 3.60 | 51 | 0.3 | 4.93 | 24 | 0.59 | 9.88 |
|  | CGA | 80 | 0.9 | 14.95 | 142 | 1.14 | 19.03 | 199 | 1.19 | 19.80 | 84 | 1.18 | 19.63 | 228 | 0.82 | 13.59 | 139 | 0.41 | 6.76 | 133 | 0.77 | 12.86 | 29 | 0.72 | 11.93 |
|  | CGG | **131** | **1.47** | **24.49** | 57 | 0.46 | 7.64 | 202 | 1.21 | 20.10 | 38 | 0.53 | 8.88 | 285 | 1.02 | 16.98 | 77 | 0.22 | 3.75 | 62 | 0.36 | 6.00 | 16 | 0.4 | 6.58 |
|  | **AGA** | 88 | 0.99 | 16.45 | **230** | **1.85** | **30.83** | 136 | 0.81 | 13.53 | **159** | **2.23** | **37.15** | **475** | **1.7** | **28.31** | **1175** | **3.43** | **57.18** | **487** | **2.83** | **47.10** | **85** | **2.1** | **34.98** |
|  | AGG | 102 | 1.14 | 19.07 | 115 | 0.92 | 15.42 | 139 | 0.83 | 13.83 | 98 | 1.37 | 22.90 | 336 | 1.2 | 20.02 | 554 | 1.62 | 26.96 | 228 | 1.32 | 22.05 | 64 | 1.58 | 26.34 |
|  |  |  |  |  |  |  |  |  |  |  |  |  |  |  |  |  |  |  |  |  |  |  |  |  |  |
| **T** | ACU | 119 | 0.92 | 23.06 | **401** | **1.49** | **37.30** | 321 | 0.9 | 22.54 | 169 | 1.06 | 26.61 | 572 | 1.19 | 29.75 | 450 | 0.94 | 23.54 | **582** | **1.92** | **47.98** | **71** | **0.98** | **24.57** |
|  | ACC | **161** | **1.25** | **31.20** | 274 | 1.02 | 25.49 | **731** | **2.05** | **51.33** | 168 | 1.06 | 26.46 | **853** | **1.77** | **44.36** | 362 | 0.76 | 18.93 | 222 | 0.73 | 18.30 | 81 | 1.12 | 28.03 |
|  | ACA | 138 | 1.07 | 26.74 | 320 | 1.19 | 29.77 | 259 | 0.73 | 18.19 | **214** | **1.35** | **33.70** | 310 | 0.64 | 16.12 | **967** | **2.02** | **50.58** | 369 | 1.22 | 30.42 | 83 | 1.15 | 28.72 |
|  | ACG | 98 | 0.76 | 18.99 | 80 | 0.3 | 7.44 | 113 | 0.32 | 7.94 | 84 | 0.53 | 13.23 | 188 | 0.39 | 9.78 | 133 | 0.28 | 6.96 | 40 | 0.13 | 3.30 | 54 | 0.75 | 18.69 |
|  |  |  |  |  |  |  |  |  |  |  |  |  |  |  |  |  |  |  |  |  |  |  |  |  |  |
| **A** | GCU | 159 | 0.8 | 20.03 | **419** | **1.39** | **34.80** | 275 | 0.78 | 19.42 | 148 | 1.09 | 27.31 | 419 | 0.9 | 22.55 | 462 | 0.95 | 23.79 | **488** | **1.7** | **42.58** | 67 | 0.86 | 21.41 |
|  | GCC | **296** | **1.49** | **37.28** | 344 | 1.14 | 28.57 | **792** | **2.24** | **55.93** | 147 | 1.08 | 27.12 | **832** | **1.79** | **44.78** | 345 | 0.71 | 17.77 | 250 | 0.87 | 21.82 | 77 | 0.98 | 24.60 |
|  | GCA | 148 | 0.75 | 18.64 | 341 | 1.13 | 28.32 | 230 | 0.65 | 16.24 | **194** | **1.43** | **35.79** | 419 | 0.9 | 22.55 | **983** | **2.02** | **50.62** | 363 | 1.27 | 31.68 | **111** | **1.42** | **35.46** |
|  | GCG | 191 | 0.96 | 24.06 | 100 | 0.33 | 8.31 | 119 | 0.34 | 8.40 | 53 | 0.39 | 9.78 | 188 | 0.4 | 10.12 | 152 | 0.31 | 7.83 | 45 | 0.16 | 3.93 | 58 | 0.74 | 18.53 |
|  |  |  |  |  |  |  |  |  |  |  |  |  |  |  |  |  |  |  |  |  |  |  |  |  |  |
| **G** | GGU | 97 | 0.55 | 13.78 | 235 | 0.82 | 20.61 | 177 | 0.62 | 15.54 | 103 | 0.77 | 19.18 | 243 | 0.53 | 13.33 | 251 | 0.4 | 9.89 | 248 | 0.84 | 20.98 | 71 | 0.88 | 22.12 |
|  | GGC | 161 | 0.91 | 22.87 | 213 | 0.75 | 18.68 | **340** | **1.19** | **29.85** | 93 | 0.69 | 17.32 | 315 | 0.69 | 17.28 | 327 | 0.52 | 12.88 | 153 | 0.52 | 12.94 | 38 | 0.47 | 11.84 |
|  | **GGA** | 188 | 1.07 | 26.70 | **466** | **1.64** | **40.88** | 312 | 1.1 | 27.39 | **236** | **1.76** | **43.95** | **682** | **1.5** | **37.41** | **1320** | **2.08** | **51.99** | **562** | **1.9** | **47.55** | **115** | **1.43** | **35.83** |
|  | GGG | **258** | **1.47** | **36.65** | 226 | 0.79 | 19.82 | 310 | 1.09 | 27.22 | 105 | 0.78 | 19.55 | 583 | 1.28 | 31.98 | 641 | 1.01 | 25.25 | 219 | 0.74 | 18.53 | 97 | 1.21 | 30.22 |
|  |  |  |  |  |  |  |  |  |  |  |  |  |  |  |  |  |  |  |  |  |  |  |  |  |  |

^#^Non-degenerate amino acids (W, M) and stop codons are excluded. The most frequently observed codons are in bold faced and among them codons that are underlined observed highest among them.
